# Predictors of uncircumcised primary school girls’ intention to genital cutting in South Ethiopia: Application of theory of planned behavior

**DOI:** 10.1371/journal.pone.0270738

**Published:** 2022-06-30

**Authors:** Solomon Haile, Dawit Sullamo, Tekle Ejajo, Firanbon Teshome, Yohannes Kebede

**Affiliations:** 1 Department of Public Health, College of Medicine and Health Science, Wachemo University, Hosanna, Ethiopia; 2 Department of Health, Behavior and Society, Faculty of Public Health, Jimma University, Jimma, Ethiopia; University of Salamanca, SPAIN

## Abstract

**Background:**

Female genital cutting (FGC) is still among the most common harmful traditional practices, especially in Africa and Asia. Despite the existence of rich evidence on the prevalence of FGC, information about the primary target audiences’ (girls’) intention toward FGC is limited. Therefore, this study aimed to identify the predictors of primary school girls’ behavioral intention toward female genital cutting.

**Methods:**

An institution-based cross-sectional study was conducted from May 08 to 28, 2019 in Dunna district, South Ethiopia. A two-stage sampling technique was used to select 354 uncircumcised female students. A self-administered pre-tested structured questionnaire was used for data collection. Data were entered into Epi data manager version 4.0.2 and exported to STATA version 16.0. Descriptive analyses such as frequency, percentage, mean and standard deviation were performed as necessary. Univariable and multivariable linear regression analyses were conducted to identify predictors of primary school girls’ intention to experience FGC.

**Results:**

The mean age of the respondents was 13.09±1.69 years with an age range of 10 to 18 years. Of the 354 respondents, 156 (44.1%) intended to experience FGC. The model accounted for 76.58% of the variance in primary school girls’ intention to experience FGC. Among socio-demographic characteristics, mothers’ educational level of ≤8 grade (β = 1.95, p<0.001) and the age of the respondents (β = -0.23, p = 0.036) predicted primary school girls’ intention toward FGC. Among the constructs of the theory of planned behavior (TPB), direct perceived behavioral control (β = 0.47, p = 0.015), indirect perceived behavioral controls (β = 0.05, p = 0.002), and direct subjective norms (β = 0.18, p = 0.039) predicted primary school girls’ intention to experience FGC.

**Conclusions:**

In this study, we found that primary school girls’ intention toward FGC was high. The educational level of mothers and the age of the respondents had a great influence on primary school girls’ intention to experience FGC. Perceived lack of power and social pressure also predicted primary school girls’ intention to engage in genital cutting. The findings suggest that FGC is mainly performed by the decision of the parents irrespective of the girls’ preferences. Therefore, behavioral change communication interventions such as media campaigns, peer education and community dialogue guided by the TPB for empowering girls to confront FGC and reducing the influence of referents need to be focused.

## Background

A female genital cutting (FGC) comprises all procedures that involve fractional or total elimination of the external genitalia or any injury to the female genital organs for cultural or other non-medical reasons [1]. FGC is a harmful traditional practice with no health benefits and is a fundamental violation of the human rights of women and girls [1]. It has four main categories: Type 1 (clitoridectomy) involves partial or total removal of the clitoral glans and/or the clitoral hood. Type 2 (Sunna) is a partial or total removal of the clitoral glans and labia minora, with or without the removal of the labia majora. Type 3 (infibulation) involves lessening the vaginal hole by creating a covering seal that can be formed by cutting and repositioning the labia minora, or labia majora, sometimes through stitching, with or without removal of the clitoral hood and glans. Type 4 includes all other forms of non-therapeutic genital alterations such as pricking, piercing, incising, scraping, and cauterizing the genital area [2, 3].

FGC is a global concern and is practiced worldwide [4, 5]. However, it is predominantly practiced and deeply rooted in social norms in over 28 African countries, with most concentrated in sub-Saharan Africa [6]. Over 200 million women and girls with FGC live in African countries [7]. FGC is also practiced in some Asian countries such as India, Indonesia, Iraq, and Pakistan [8–10]. Evidence shows that more than half of women and girls with FGC live in Indonesia, Egypt, and Ethiopia [7]. FGC is practiced by over half of Ethiopia’s ethnic groups, and it is a common harmful traditional practice that highly affects women and children [11]. In Ethiopia, more than 6 out of 10 women experience FGC [12]. The prevalence of FGC was higher than the national prevalence in some regions of the country. For instance, it was 99% in the Somali region [12], 88.1% in the Kersa district, East Hararge [8], and 82.2% in the Hadiya zone [13]. The prevalence of FGC varies among age groups. For instance, 15.7% of girls aged 0 to 14 years experienced FGC in Ethiopia [12]. Another study from the same area showed that the prevalence of FGC among girls aged 0 to 14 years was 18.6% [14]. Among the circumcised girls, 66%, 26%, and 7% were circumcised at the age of 0 to 4 years, 5 to 9 years, and 10 to 14 years, respectively [14]. Half of the girls within the age groups of 15 to 19 years in the Southern Nations, Nationalities, and Peoples’ region (SNNPR) have undergone FGC [15].

FGC is practiced for various reasons such as maintaining cleanliness, lessening female sexual pleasure thereby reducing promiscuity and extramarital sexual acts, protecting virginity, preparing the girl for marriage, family’s honor, increasing social acceptance and religious support, enhancing fertility, perceived safe delivery of babies, and promotion of child survival [16–20]. This indicates how deeply FGC has been rooted in communities. FGC has serious negative consequences such as physical, social, economic, and psychological impacts on women and young girls, especially for those who undergo extreme forms of the procedure [21–23]. The health consequences of FGC can be immediate or long-term. The immediate health consequences include severe pain, hemorrhage, genital tissue swelling, episiotomy, perineal tear, infections (HIV/AIDS, hepatitis, tetanus, sepsis, and acute urinary retention), bone fractures, shock, and death [24–27]. The long-term health consequences of FGC include chronic pain, psychological disturbances, painful urination, repeated urinary tract infections, vaginal discharge, vaginal itching, pain and difficulty in passing menstrual blood, pain during sexual intercourse, decreased sexual satisfaction, increased risk of childbirth complications, cesarean delivery, fistula, risk of surgery (de-infibulation), increased risk of stillbirth, early neonatal death and neurological deficits due to severe birth asphyxia [2, 24, 25, 28]. For instance, evidence shows that women who have undergone infibulation face a 70% greater risk of hemorrhage than those who don’t have any other form of FGC [29].

To eradicate FGC, different strategies such as human rights frameworks, legal mechanisms, a health risk approach, training health workers as change agents, training and converting circumcisers, the alternative rites approach, the positive deviance approach, and comprehensive social development approaches have been implemented in different countries [2, 30–32]. The Ethiopian FGC law also pointed out that FGC is illegal and its performance leads to punishments such as imprisonment for a period of not less than three months or a fine of not less than 9.62 US dollars [33]. However, enforcement of the law seems to be weak and incomprehensive, failing to protect uncut women from verbal abuse or exclusion from society and does not address cross border FGC [33].

Although efforts have been made to eradicate FGC, it commonly persists in many countries, especially in Africa, and is still far from attaining the third and fifth sustainable development goals [34]. Several studies have been conducted on the prevalence of FGC across different countries of the world and some evidence exists on parental intention to circumcise daughters [19, 35–39]. However, to the best of our knowledge, the intention of primary risk groups (girls) toward FGC is unknown. Ending FGC is unlikely without considering the opinions of the victims. Therefore, this study aimed to identify predictors of primary school girls’ intention to experience FGC in Dunna district, South Ethiopia.

## Materials and methods

### Study design, setting, and period

An institution-based cross-sectional study was conducted from May 08 to 28, 2019 among primary school uncircumcised girls in Dunna district, Hadiya zone, South Ethiopia. Dunna district is one of the districts in the Hadiya zone. The district has 32 kebeles (the lowest governmental administrative unit next to the district). Donna district has 36 primary schools. The total number of female students in the 36 primary schools were 16,475, of which 5,080 were in grade 5 to 8. Among the total of 5,080 grade 5 to 8 female students, 1,986 were uncircumcised. There were 3,156 grade 5 to 8 female students in the selected six schools, of which 475(15.1%) were uncircumcised.

### Source and study population

The source populations were all uncircumcised primary school female students in Dunna district, who were enrolled in 2019. The study population consisted of randomly selected grade 5 to 8 uncircumcised female students.

### Sample size determination

The sample size was computed using a single population proportion formula [(n = (Z1 -ά/2)^2^p(1—p)/d^2^)] considering the following assumptions: 50% proportion of intention of uncircumcised female students toward circumcision since there were no prior studies that could address the same objectives, 95% level of confidence, and 5% margin of error. This yielded a sample size of 384. As the number of uncircumcised female students (1,986) in the 36 primary schools of Dunna district was less than 10,000, the sample size was corrected. Considering a 10% non-response rate, a total of 354 respondents were included in the study.

### Sampling procedures

In this study, a two-stage sampling technique was used to select the respondents. In the first stage, 6 out of 36 primary schools with grades 5 to 8 in the Dunna district were selected using a ballot method of simple random sampling technique. In the second stage, the respondents were selected as follows: Uncircumcised female students were identified step-by-step after consulting the teachers as circumcision is a sensitive issue in Ethiopia. The anonymity of the participants was emphasized, and confidentiality was maintained. Accordingly, orientation was given for all grade 5 to 8 female students by female teachers in private classes on the objective of the study, their confidentiality, and the need to provide the right information. After orientation was given, the female teachers called the students individually to leave the class and asked them whether they had been circumcised, while the students exited the gate of the class in a manner in which they kept their confidentiality. Accordingly, a total of 1,986 uncircumcised female students were identified in the 36 primary schools of the Dunna district. Among the 1,986 uncircumcised female students, 475 were found in the six selected schools and served as a sampling frame. The sample size was then proportionally allocated to the selected schools. Finally, a computer-generated simple random sampling technique was used to select the required number of study participants.

### Data collection tools and techniques

The authors developed a structured questionnaire based on the standard guidelines of the TPB [40, 41]. First, an elicitation study was conducted to identify salient beliefs underlying attitudes, subjective norms, and perceived behavioral controls related to female circumcision. Accordingly, a semi-structured interview guide was used and interviews (elicitation study) were conducted in Lemo district primary schools with ten female students. The data obtained from the interviews were then analyzed and used to develop a quantitative three-point Likert scale questionnaire for each dimension of the TPB. The questionnaire was first prepared in English and translated into the local language Hadiyisa, and back-translated into English to ensure consistency. The questionnaire had three major parts: Socio-demographic characteristics, constructs of TPB, and intention toward FGC (S1 Questionnaire). The internal consistency of the items was checked using Cronbach’s alpha. Accordingly, the alpha of direct attitude α = 0.748, direct subjective norm α = 0.773, direct perceived behavioral Control α = 0.736, intention α = 0.791, behavioral belief α = 0.801, evaluation of behavior α = 0.783, overall indirect attitude (weighted behavioral belief) α = 0.797, normative belief α = 0.725, motivation to comply α = 0.744, overall indirect subjective norm (weighted normative belief) α = 0.820, control belief α = 0.735, power of control α = 0.811, and overall indirect perceived behavioral control (weighted control belief) α = 0.715. In this study, only face and content validity was focused. Three public health experts examined the relevance, adequacy, and representativeness of the questionnaire items. In addition, during pretesting, respondents were invited to provide suggestions on the clarity, language, and simplicity of the items. The feedback obtained from experts and respondents was considered and modifications such as question-wording, sequence, grammar, and correction of typos were made. The other types of validity were not addressed in this study.

Six data collectors and three supervisors were recruited for data collection based on their fluency in the local language. In this study, a self-administered questionnaire was used to collect the data as circumcision is a sensitive issue in Ethiopia and females were ashamed of talking about it. Data collection took place in private rooms. For better clarity and understanding of the meaning of the questions, orientation was given to the study participants on each section of the questionnaire and how to fill them. In addition, the data collectors were with the study participants during the data collection period to clarify questions when respondents felt ambiguity with the questions, check completeness, and collect questionnaires from the respondents. When the questionnaires were incomplete, the data collectors requested the respondents to answer all questions. Supervisors checked the data collection process on a daily basis.

### Variables

The outcome variable was uncircumcised primary school girls’ intention toward FGC. The explanatory variables were the socio-demographic characteristics of the respondents and constructs of TPB: attitude (behavioral belief and evaluation of behavior), subjective norm (normative belief and motivation to comply), and perceived behavioral control (control belief and power of control).

### Measurements

#### Elicitation study

This study explored silent beliefs pertaining to the circumcision of school girls guided by constructs of the TPB. Accordingly, three categories of silent beliefs were identified: A) Behavioural beliefs (consequences of un/circumcision) such as schoolgirls being accepted by the community and free from shame when circumcised, carrying out circumcision will not lead to a criminal appeal, and uncircumcision results in poor hygiene, promiscuity, and being cursed. B) Normative beliefs (school girls’ perception of who approves of girls’ circumcision) indicated that families, siblings, neighbors, close friends, uncircumcised female friends, health care providers, and teachers are key referents for school girls’ circumcision status. C) Control beliefs (school girls’ perceived inhibitors and facilitators of circumcision) such as lack of consent of the human subject to circumcision irrespective of girls’ willingness status, loose implementation or follow-up of circumcision laws at grassroots levels, low prevalence of uncircumcision among girls, parental pre-arrangements and scheduling of the circumcision, and low self-confidence to confront influences to undergo circumcision.

#### Intention toward FGC

Intention was assessed using 7 items. Each item was scored on a three-point Likert scale with response options “1 = disagree, 2 = neutral, and 3 = agree”. In this study, intention was treated as both a continuous and categorical variable as the study aimed to determine the prevalence and predictors of the intention to experience FGC. The authors treated intention as a continuous variable to identify its predictors. The total intention score was computed by summing all seven items. A higher score indicates a higher level of intention to experience FGC. To determine the prevalence, the intention was dichotomized based on the mean score. Those who scored the mean score and above were considered “***intended to have FGC****”* and those who scored below the mean were considered “***unintended to have FGC***”.

#### Attitudes toward female circumcision

Were assessed in two ways: directly and indirectly (belief-based attitude). For direct measures, respondents were asked 3 items on a three-point Likert scale to rate their attitudes toward circumcision. The total score was then computed and a higher score indicates a more favorable attitude toward circumcision. Indirect measures of attitude (weighted behavioral belief) were constructed from a total of 16 items (8 items for behavioral beliefs regarding circumcision and 8 items for evaluation of beliefs) on a three-point Likert scale. Items of behavioral beliefs and outcome evaluation were used to compose the indirect attitude scale; where each behavioral belief item score was multiplied by its corresponding outcome evaluation item scores. Then, the items were summed to compose the belief-based attitude scale. A higher score indicates a more favorable attitude toward FGC.

#### Subjective norms

Whether the respondents thought important others wanted them to circumcise or whether the respondents felt social pressure to circumcise or think it is expected of them to circumcise. This was measured using both direct and indirect methods. The direct subjective norm was measured using 5 items on a three-point Likert scale. Then, the total score was computed by summing all 5 items. The indirect measures (weighted normative beliefs) were measured by assessing uncircumcised girls’ perception of the approval of their referents on circumcision and their motivation to comply with those referents. Twenty items (10 for normative beliefs and 10 for motivation to comply) were used to assess the indirect measure of subjective norms. The composite measure was computed by multiplying the normative belief score with the corresponding motivation to comply. Finally, the total score was obtained by summing all the items. The higher the score, the higher the approval of referents for girls’ circumcision.

#### Perceived behavioral control

Direct perceived behavioral control was assessed by asking respondents to rate the extent to which they would be able to resist or accept female circumcision. It was measured using 3 items on a three-point Likert scale, and a total score was obtained by summing up all the items. A higher score indicates a higher acceptance of female circumcision. Eighteen items (9 for control belief and 9 for power to control) were used to assess indirect measures of perceived behavioral control. Weighted control scores were computed by multiplying the control belief score by the corresponding perceived power items. Finally, the weighted items were summed to yield the total score. In this study, the higher the score of perceived behavioral control, the higher one’s belief in the difficulty of overcoming or confronting FGC. This means that there is a lack of power to resist the FGC. A three-point Likert scale with the response options “1 = disagree, 2 = neutral, and 3 = disagree” was used to measure all the constructs of the TPB.

### Data quality assurance

The questionnaire was first prepared in English and then translated into the local language and back to English to ensure consistency. Before the commencement of data collection, a one-day intensive training was provided for data collectors and supervisors on the objective of the study, data collection tools, data collection techniques, handling ethical issues, and maintaining the confidentiality and privacy of the respondents. A pre-test was conducted on 5% of the sample size and some modifications such as question-wording, sequence, grammar, and correction of typos were made based on the findings. Female teachers were recruited to identify uncircumcised girls. All the data collectors were female to boost the confidence of the respondents. This allowed respondents to ask questions that were unclear to them. The Likert scale questionnaire was reduced to three points for each dimension of the TPB considering the age of the study participants for a better understanding of the questions. The collected data were checked daily for completeness and consistency.

### Data processing and analysis

Data were entered into Epidata version 4.0.2. It was then exported to STATA version 16.0 (S1 Data). Descriptive statistical measures such as frequency, percentage, mean and standard deviation were calculated. A factor analysis using principal component analysis (PCA) was conducted for data reduction. Pearson’s correlation coefficient was used to determine correlations between the items. Inter-item correlation (r> 0.30) and Item-total correlation r> 0.50) were observed. Cronbach’s alpha (α ≥0.7) was used to indicate that the scale items were internally consistent in the variable to which they belonged [42]. Univariable and multivariable linear regression analyses were conducted to identify predictors of uncircumcised primary school girls’ intention toward FGC. Univariable regression models were first built for each explanatory variable with the primary school girls’ behavioral intention scores. Accordingly, 12 variables (age of the respondents, residence, history of sister’s circumcision, history of circumcision in their neighbors, grade of the respondents, mothers’ educational status, direct attitude, direct subjective norms, direct perceived behavioral control, indirect attitude, indirect subjective norms and indirect perceived behavioral controls) were screened. Then, 9 variables with a p-value <0.25 (age of the respondents, history of sisters’ circumcision, mothers’ educational status, direct attitude, direct subjective norms, direct perceived behavioral control, indirect attitude, indirect subjective norms, and indirect perceived behavioral controls) were fitted into multivariable linear models for analysis [43]. Multivariable linear regression analysis was conducted using a stepwise backward elimination technique to control for confounders and identify the independent effects of each explanatory variable on the outcome variable [44]. Variables with p-values <0.05 were recognized as significant predictors of respondents’ intention toward FGC. The assumptions of the linear regression were checked and no violations were present.

### Ethical considerations

This study was approved by the Institutional Review Board (IRB) of Wachemo University. Letter of ethical approval for data collection was given by Apr 22, 2019, with the reference number ማ/ሳ/ቁ/ሣ/ቱ/ም/ጦ/ቶ 019/11. The necessary permission was obtained from the Dunna district education offices, the headmasters of the schools, and the instructors of the selected classes to recruit the participants and collect data. The researcher discussed ethical issues with school officials and that the study had no harm to the students, their families, or school environments. All participants were informed of the purpose and benefits of the study. Written assent was obtained from each participant. They were informed that participation in the study is voluntary and that they can refuse to participate or withdraw from the study without any penalties. Moreover, consent to participate in the study was secured from the participants’ parents or guardians, and confidentiality of the information was ensured.

## Results

### Socio-demographic characteristics of the respondents

Table 1 shows the socio-demographic characteristics of the respondents. The mean age of the respondents was 13.09±1.69 years with an age range of 10 to 18 years. The majority, 249(70.3%) and 270(76.3%) of the respondents were from rural areas by residency and protestants by religion, respectively. In addition, a majority, 237(67.0%) and 219(61.9%) had a history of their sister circumcision and history of circumcision in their neighbors by this year, respectively (Table 1).

**Table 1:** Socio-demographic characteristics of primary school female students in Dunna district, South Ethiopia, 2019.

Variable	Category	Frequency (N = 354)	Percent
Age of the respondents	Mean(±SD)	13.09(±1.69)	
Residence	Urban	105	29.7
	Rural	249	70.3
Grade of students	Grade 5	106	29.9
	Grade 6	86	24.3
	Grade 7	93	26.3
	Grade 8	69	19.5
Religion	Orthodox	76	21.5
	Protestant	270	76.3
	*Others	8	2.26
Mothers’ education	Can’t read and write	103	29.1
	Can read and write	112	31.6
	Grade 1–8	75	21.2
	Grade 9–12	32	9.0
	Collage and above	32	9.0
Hx of sister’s circumcision	Yes	237	67.0
	No	117	33.0
Hx of circumcision in their neighbors this year	Yes	219	61.9
	No	135	38.1

*Muslim, Catholic; Hx: History

### Intention toward female genital cutting

The majority, 250(70.6%) of the respondents disagreed with the question that asked I will be circumcised in the next 1 year. Similarly, 248(70.1%) didn’t want to encourage their friends and sisters to have circumcision or intended to inform legal bodies if any circumcision occurred in their neighbors. The study also found that about 156 (44.1%) of them intended to experience FGC in the following year (Table 2).

**Table 2:** Behavioural intention toward female genital cutting among primary school girls in Dunna district, South Ethiopia, 2019 (N = 354).

**Items of behavioral intention**	**Disagree**	**Neutral**		**Agree**
I have a willingness to circumcision in the next 1 year	173(48.9)	37(10.5)		144(40.7)
I have a readiness for circumcision in the next 1 year	181(51.1)	43(12.1)		130(36.7)
I have an intention to circumcision in the next 1 year	176(49.7)	55(15.5)		123(34.7)
I will be circumcised in the next 1 year	250(70.6)	38(10.7)		66(18.6)
I want to encourage my friends and sisters to have circumcision	248(70.1)	21(5.9)		85(24.0)
I want to discuss with others on circumcision issue	237(66.9)	33(9.3)		84(23.7)
I have no intention to inform legal bodies if any circumcision attempt is made on me	248(70.1)	26(7.3)		80(22.6)
**Intention towards FGC**	**Unintended**		**Intended**	
	198 (55.9%)		156 (44.1%)	

### Direct measures: Constructs of TPB

As shown in Table 3, more than half 196(55.4%) and 190(53.7%) of the respondents disagreed that female circumcision was good and important, respectively. The findings also showed that 257(72.6%) of the respondents agreed that circumcised girls wanted uncircumcised girls to undergo circumcision. Similarly, the majority, 300(84.7%), 274(77.4%), and 272(76.8%) of the respondents believed that having circumcision was beyond their control, resisting circumcision was difficult for them and they agreed that they had no full confidence in not experiencing circumcision, respectively (Table 3).

**Table 3:** Frequency of direct constructs of TBP among primary school girls in Dunna district, South Ethiopia, 2019 (N = 354).

**Direct Attitude**	**Disagree**	**Neutral**	**Agree**
Female circumcision is good	196(55.4)	121(34.2)	37(10.5)
Female circumcision is important	190(53.7)	128(36.2)	36(10.2)
Female circumcision is pleasant	165(46.6)	31(8.8)	158(44.6)
**Direct Subjective Norm**	**Disagree**	**Neutral**	**Agree**
Circumcised girls want me to undergo circumcision	74(20.9)	23(6.5)	257(72.6)
Most of my referents expect me to have circumcision	73(20.6)	82(23.2)	199(56.2)
Most of my referents want me to experience circumcision	88(24.9)	81(22.9)	185(52.3)
Most people who are important to me inspire for my circumcision	77(21.8)	96(27.1)	181(51.1)
Most people who are important to me like my circumcision	108(30.5)	92(26.0)	154(43.5)
**Direct Perceived Behavioural Control**	**Disagree**	**Neutral**	**Agree**
I believe that having circumcision is beyond my control	14(4.0)	40(11.3)	300(84.7)
Resisting circumcision is difficult to me	18(5.1)	62(17.5)	274(77.4)
I am not fully confident about not having circumcision	18(5.1)	64(18.1)	272(76.8)

### Indirect measures: Constructs of TPB

#### A) Indirect attitude

Regarding behavioral beliefs, 211(59.6%) of the respondents disagreed that female circumcision had no criminal effect. However, 191(54.0%) and 229(64.7%) of the respondents agreed that being uncircumcised was culturally unacceptable, and led the female to be cursed by the community, respectively. Regarding the evaluation of the behavior; 231(65.3%) of the respondents preferred circumcision over being insulted by others, 227(64.1%) agreed that circumcision was better than feeling shame, and 214(60. 5%) preferred circumcision rather than losing acceptance by the community (Table 4).

**Table 4:** Descriptive statistics of indirect attitude regarding female genital cutting among primary school girls in Dunna district, South Ethiopia, 2019 (N = 354).

**Items of behavioral beliefs**	**Disagree**	**Neutral**	**Agree**
Female circumcision has no criminal effect	211(59.6)	17 (4.8)	126(35.6)
Uncircumcision of females results in a feeling of uncleanness	113(31.9)	26(7.3)	215(60.7)
The uncircumcised females have promiscuity behavior	156(44.1)	33(9.3)	165(46.6)
Female circumcision has no health problems	150(42.4)	21(5.9)	183(51.7)
Uncircumcision let females to be insulted by others	136(38.4)	28 (7.91)	190(53.7)
Uncircumcision of females is shameful	133(37.6)	24 (6.8)	197(55.7)
Uncircumcision is culturally unacceptable	134(37.9)	21 (5.9)	199(56.2)
Uncircumcision lead females to be cursed by community	109(30.8)	16 (4.5)	229(64.7)
**Evaluation of behavior**	**Disagree**	**Neutral**	**Agree**
Criminalization of female circumcision is not good	170(48.0)	12(3.4)	172(48.6)
Circumcision is better than lack of neatness	167(47.2)	21(5.93)	166(46.9)
Circumcision is preferable to having promiscuity	157(44.4)	39(11.0)	158(44.6)
Better to face health problems due to circumcision than to stay uncircumcised	126(35.6)	32(9.0)	196(55.4)
Circumcision is preferable than being insulted by others	106(29.9)	17 (4.8)	231(65.3)
Circumcision is better than feeling shame	103(29.1)	24(6.8)	227(64.1)
Circumcision is better than losing acceptance by the community	121(34.2)	19(5.4)	214(60.5)
I believe in being circumcised than being cursed by the community	96 (27.1)	34(9.6)	224(63.3)

#### B) Indirect subjective norms

As shown in Table 5, among the 354 respondents, 258(72.9%) and 239(67.5%) agreed that their fathers and mothers expected them to be circumcised, respectively. Nearly half, 178(50.3%) of the respondents disagreed that their close friends and teachers supported their circumcision. Regarding the motivation to comply, the majority of primary school girls’ referents approved their circumcision. Two hundred and forty-three (68.6%), 224(63.3%), and 221(62.4%) of the respondents agreed that their father, mother, and sister/brother approved of their circumcision, respectively. However, the majority, 231(65.3%) of the respondents disagreed that their neighbors approved of their circumcision (Table 5).

**Table 5:** Descriptive statistics of indirect subjective norms among primary school girls in Dunna district, South Ethiopia, 2019 (N = 354).

**Normative beliefs**	**Disagree**	**Neutral**	**Agree**
My mother expects me to be circumcised	7(2.0)	108(30.5)	239(67.5)
My father thinks that I have to be circumcised	4 (1.1)	92(26.0)	258(72.9)
My neighbours support my circumcision	173(48.9)	71(20.1)	110(31.1)
My close friends support my circumcision	178(50.3)	55(15.5)	121(34.2)
My sister or brother expect me to have circumcision	13(3.7)	104(29.4)	237(67.0)
My health extension workers expect me to have circumcision	135(38.1)	19(5.4)	200(56.5)
My teachers expect me for having circumcision	178(50.3)	21(5.9)	155(43.8)
Government officials expect me to have circumcision	124(35.03)	22 (6.2)	208(58.8)
Uncircumcised girls expect me for circumcision	1180(33.3)	39(11.0)	197(55.7)
Circumcised girls expect me for having circumcision	2 (0.6)	122(34.5)	230(65.0)
**Motivations to comply**	**Disagree**	**Neutral**	**Agree**
My mother approves of my circumcision	5 (1.4)	125(35.3)	224(63.3)
My father approves of my circumcision	2(0.6)	109(30.8)	243(68.6)
My neighbours approve of my circumcision	231 (65.3)	31(8.8)	92 (26.0)
I would like to do what my close friends expect me to do	192 (54.2)	30(8.5)	132(37.3)
I would like to do what my sister or brother expects me to do	7(2.0)	126(35.6)	221(62.4)
My health extension workers approve of my circumcision	145(41.0)	21(5.9)	188(53.1)
My teachers approve of my uncircumcision	126(35.6)	21(5.9)	207(58.5)
I would like to do what government officials expect me to do	116(32.8)	17(4.8)	221(62.4)
Uncircumcised girls approve of my circumcision	129 (36.4)	17(4.8)	208(58.8)
Circumcised girls approve of my circumcision	5(1.4)	122(34.5)	227(64.1)

#### C) Indirect perceived behavioral control

As shown in Table 6, the control beliefs and power of the controls were assessed using a three-point Likert scale. Findings showed that the majority, 253(71.5%) of the respondents agreed that their family couldn’t give them consent for being uncircumcised. However, the majority, 216(61.0%) of respondents disagreed that information they have about circumcision is less likely to save them from circumcision. The power of control results showed that nearly three quarters, 260(73.4%) of the respondents agreed that the absence of uncircumcised girls in their neighborhood made their decision to stay uncircumcised difficult (Table 6).

**Table 6:** Descriptive statistics of indirect perceived behavioral controls among primary school girls in Dunna district, South Ethiopia, 2019 (N = 354).

**Control beliefs**	**Disagree**	**Neutral**	**Agree**
To be uncircumcised, I can’t get the consent of human subjects from family	75 (21.2)	26(7.3)	253(71.5)
Even though I do not want, my family can enforce me to have circumcision	132(37.3)	38(10.7)	184(52.0)
My family members pre-arrange and schedule the circumcision	88(24.9)	41(11.6)	225(63.6)
The high prevalence of circumcised girls in my neighborhood has a great influence on me to have circumcision	8(2.3)	93(26.3)	253(71.5)
Even though the law discourages circumcision, it can’t save me from circumcision	162(45.8)	12(3.4)	180(50.8)
Communities’ lack of information about the negative consequences of circumcision puts pressure on me to be circumcised	111(31.4)	42(11.9)	201(56.8)
The high prevalence of circumcised school girls makes my decision hard	192(54.2)	28(7.9)	134(37.9)
The information I have about the disadvantages of uncircumcision is less likely to save me from circumcision	216(61.0)	28(7.9)	110(31.1)
My being under family makes my decision on circumcision hard	157(44.4)	21(5.9)	176(49.7)
**Power of control**	**Disagree**	**Neutral**	**Agree**
My family does not allow me to the uncircumcision	146(41.2)	38(10.7)	170(48.0)
I fail to resist having circumcision if my family enforces me	127(35.9)	28(7.9)	199(56.2)
Pre-arrangement and scheduling of circumcision by my family members make my decision difficult	143(40.4)	20(5.6)	191(54.0)
The absence of uncircumcised girls in my neighbor makes my decision hard	4(1.1)	90(25.4)	260(73.4)
Loose implementation or follow-up of circumcision laws makes my decision difficult	157(44.4)	26(7.3)	171(48.3)
Communities’ lack of awareness about the consequences of circumcision makes my decision difficult	148 (41.8)	34 (9.6)	172(48.6)
It is difficult to have the power of resisting circumcision due to the high prevalence of circumcised school girls	143(40.4)	22(6.2)	189(53.4)
The information I have about circumcision may not realize my decision	100(28.2)	18(5.1)	236(66.7)
Being under my family will let me to experience circumcision	105(29.7)	37(0.5)	212(59.9)

### Mean score and standard deviation of the constructs of TPB

Table 7 shows the mean scores and standard deviations of the TPB constructs. Among the direct constructs of TPB, direct perceived behavioral controls had the highest standardized mean score (2.75). Among the indirect constructs of TPB, indirect subjective norms had the highest standardized mean score (5.32) followed by indirect perceived behavioral controls (4.84).

**Table 7:** Frequency distribution of mean scores, standard deviations, and standardized mean of the constructs of TPB among primary school girls in Dunna district, South Ethiopia, 2019 (N = 354).

Components	No. of items	Min.	Max.	Mean	Standard Deviation	Standardized Mean
Direct attitude	3	3	9	6.90	1.57	2.30
Direct SN	5	5	15	11.57	2.61	2.31
Direct PBC	3	3	9	8.25	1.29	2.75
Indirect attitude	11	12	94	37.65	17.04	3.42
Indirect SN	10	22	90	53.17	12.73	5.32
Indirect PBC	9	12	81	43.57	14.03	4.84
Intention	7	7	21	11.74	4.07	1.68

SN: Subjective norm; PBC: Perceived behavioral control

#### Predictors of respondents’ intention towards female genital cutting

In this study, the model accounted for 76.58% of the variance in primary school girls’ intention toward FGC. After controlling for confounding factors; the mothers’ educational level, age of the respondents, direct subjective norm, direct perceived behavioral controls, and indirect perceived behavioral controls were predictors of primary school girls’ intention to experience FGC. Findings showed that students who had mothers who attended grade 8 or less (adjusted β = 1.95; 95% CI 0.90 to 3.01; p<0.001) had higher intention toward FGC than those whose mothers attended education levels above grade eight. The findings also showed that the respondents’ age was associated with their intention to experience FGC. For a one-year increase in the age of the respondents, their intention to experience FGC decreases by 0.23 (adjusted β = -0.23; 95% CI -0.45 to -0.01; p = 0.036) provided that the other conditions did not vary. Primary school girls’ referents determine their intention toward circumcision. For a positive unit change in referents’ approval of circumcision, primary school girls’ intention toward FGC was increased by 0.18 units (adjusted β = 0.18; 95%CI 0.01 to 0.34; P = 0.039) given that other variables were kept constant. The findings also showed that a lack of power most predicted primary school girls’ intention toward FGC. For a positive unit change in direct and indirect perceived behavioral control, intention toward FGC increased by 0.47 units (adjusted β = 0.47; 95% CI 0.09 to 0.85; p = 0.015) and by 0.05 units (adjusted β = 0.05 95% CI 0.02 to 0.08; P = 0.002), respectively (Table 8). The findings of this study indicated that among items of indirect perceived behavioral controls, respondents’ perceptions of the high prevalence of circumcised girls in their neighbors and school made their decision not to stay uncircumcised hard.

**Table 8:** Predictors of intention towards female genital cutting among primary school girls in Dunna district, South Ethiopia, 2019 (N = 354).

Variable	Intention towards experiencing FGC					
	Univariable regression			Multivariable regression		
	β	[95% CI]	P-value	β	[95% CI]	p-value
Mothers’ educational level						
≤ grade 8	2.03	[0.94 to 3.11]	<0.001	1.95	[0.90 to 3.01]	<0.001
> grade 8	Ref.			Ref.		
Hx of sister circumcision						
Yes	0.59	[-0.31 to 1.49]	0.199	0.57	[-0.29 to 1.44]	0.191
No	Ref.			Ref.		
Direct Attitude	0.30	[0.034 to 0.57]	0.027	0.10	[-0.18 to 0.38]	0.481
Indirect Attitude	0.02	[-0.01 to 0.04]	0.207	0.02	[-0.01 to 0.04]	0.254
Direct SN	0.26	[0.10 to 0.42]	0.002	0.18	[0.01 to 0.34]	0.039
Indirect SN	0.02	[-0.01 to 0.05]	0.214	0.01	[-0.03 to 0.04]	0.682
Direct PBC	0.66	[0.27 to 1.04]	0.001	0.47	[0.09 to 0.85]	0.015
Indirect PBC	0.06	[0.03 to 0.09]	<0.001	0.05	[0.02 to 0.08]	0.002
Age of respondents	-0.19	[-0.42 to 0.03]	0.096	-0.23	[-0.45 to -0.01]	0.036

## Discussion

In this study, 156 (44.1%) of respondents intended to experience FGC within the next 1 year. Mothers’ educational status, age of the respondents, direct subjective norms, direct perceived behavioral controls, and indirect perceived behavioral controls had statistically significant associations with primary school girls’ intention to experience FGC.

In the current study, among 3,156 grade 5 to 8 female students, only 475 (15.1%) were uncircumcised. This implies a rate of FGC of 85% prior to grade 5. The current study was higher than the previous studies from Ethiopia [12, 14, 15]. This may be due to the possibility that many girls declare themselves as circumcised despite not being circumcised to avoid carrying out the survey in the current study. In addition, the discrepancy may also be due to the current study being conducted in a small area. Indeed, the inconsistency may also be due to differences in the source of information. In previous studies, mothers were interviewed to answer the circumcision status of their daughters. However, in the current study, the direct victims (girls) participated.

This study indicated that 156 (44.1%) of the respondents had the intention to experience FGC and more than half, 198 (55.9%) of the respondents had no intention of getting FGC within the following year. School girls’ intention to undergo FGC implies how FGC is common and deep-rooted in the area due to factors other than a lack of awareness about its disadvantages or negative consequences. This may be due to the intention of parents or communities to circumcise women and girls. The community didn’t accept uncircumcision and cursed girls for being uncircumcised. Even though it varies from region to region, evidence from Ethiopia showed that mothers were highly intent to circumcise their daughters. For instance, a cross-sectional study from the Somali regional state in Eastern Ethiopia indicated that more than eight out of ten mothers (84%) had the intention of circumcising their daughters [45]. A study from the Bale Zone, Ethiopia also showed that more than one-fourth (26.7%) of women intend to continue FGC [19]. These findings suggest the need for designing social and behavioral change communication interventions both at schools for students and at the community level for parents and other referents.

The current study also identified predictors of primary school girls’ intention to experience FGC. The findings showed that, of the constructs of TPB, direct perceived behavioral control strongly predicted primary school girls’ intention to experience FGC. For a unit increase in scores of direct perceived behavioral controls, the primary school girls’ intention to experience FGC increased by 0.47 provided that the other conditions were unvaried. This indicates that primary school girls lack the power to control circumcision. As the respondents’ perceived difficulty in overcoming circumcision increases, their intention to experience FGC also increases. This finding suggests the need for behavior change communication interventions for students to boost their confidence in resisting circumcision. For instance, by informing legal bodies for any circumcision attempts made on them or other females. The current study also showed an association between control beliefs and intention to experience FGC. For a unit increase in scores of indirect perceived behavioral controls, the primary school girls’ intention to experience FGC increases by 0.05 provided that other variables remain constant. Among the items of indirect perceived behavioral controls, primary school girls’ perception of the high prevalence of circumcised girls in their neighbors and schools increased their lack of power to confront circumcision and intent them toward FGC. This implies the need for interventions that focus on controlling facilitators of FGC (e.g., preventing fallacious information about the benefits of circumcision) and strengthening inhibitors (e.g. circumcision laws) to avoid performing FGC. Evidence from previous studies also showed that perceived behavioral controls had a significant association with mothers’ intention to perform FGC on daughters [35, 38].

The present study also showed a significant association between perceived social pressure and primary school girls’ intention to experience FGC. For one unit increase in direct perceived subjective norms, the primary school girls’ intention towards experiencing FGC increases by 0.18 provided that other variables are kept constant. The respondents were perceiving that their referents think they need to be circumcised. This suggests that FGC is mainly performed by the decision of the parents irrespective of the girls’ preferences. This is consistent with the assumption of the TPB that when subjective norm and perceived control become positive toward the behavior, the likelihood of performing the behavior is high [40, 41]. Previous studies also showed that subjective norms had a significant association with women’s intention to perform FGC on daughters [38]. This implies the need for behavioral change communication interventions such as communication campaigns on the negative health implications of FGC both for students as well as for their referents. Evidence showed that multimedia communication program has been effective in changing and promoting the intention not to perform FGC [46]. In the current study, the attitude had no significant association with primary school girls’ intention to have FGC. That means the respondents had no positive affective and instrumental evaluations of experiencing genital cutting. This implies the respondents’ intent of having FGC was due to perceived external factors than internal positive feelings towards genital cutting.

In the current study, among the socio-demographic factors, mothers’ educational level and the age of the respondents had a statistically significant association with primary school girls’ intention to experience FGC. Students who had mothers attended education of grade 8 or less had the intention of experiencing FGC in the next year. This implies that there was a disparity of intending FGC among female students concerning their mothers’ educational level, which indicates empowering women through education is very important to combat FGC and reduce its negative consequences. In addition, this underscores the need to design health education programs and conduct information, education, and communication interventions among mothers on FGC, especially on its negative health implications. This finding was similar to studies conducted in Iran [38], Sierra Leone [37], Nigeria [35], and Ethiopia [19, 47], which showed that women’s intention not to perform FGC on daughters increases with their educational level. We also found that the age of the respondents predicted their intention to experience FGC. For one year increase in age of the respondents, their intention to experience FGC decreases by 0.23 keeping other variables constant. This might be due to a selection effect. In the current study, females who survived without FGC were selected as a sample, and they are less likely to experience FGC. Girls who survive the higher risk ages without FGC are more likely to belong to the less at-risk group since they survived the risky ages without mutilation. This study has several strengths and limitations. In this study, the concept of the direct and indirect constructs of the TPB was well utilized in determining the primary school girls’ intention toward FGC. The other strength of the present study was that, to the best of the authors’ knowledge, this was the first study that measured the primary risk groups’ (girls’) intention towards FGC, unlike previous studies which focused on mothers’ intention towards circumcising their daughters. This study also has several limitations. The potential limitation was that there might be the possibility of missing uncircumcised girls as the identification was based on students’ verbal reports and the issue is very sensitive. There was also the possibility that many girls declared themselves as circumcised despite not being circumcised to avoid carrying out the survey. Thus, the findings of the current study need to be interpreted with caution. To mitigate the possibility of under or over-reporting, female students were well informed about the purpose of the study. In addition, the identification of the uncircumcised girls was conducted by female teachers. Indeed, to keep their confidentiality, orientation was given only to female students in private classes and they were informed that the information obtained from them would not be shared with any individuals and only used for scientific communications. The other limitation was that the findings of the study may suffer from social desirability bias as the study is relatively sensitive. Furthermore, the use of a self-administered questionnaire may affect the clarity of the questions. The authors decided to use a self-administered questionnaire after weighing its cons and pros as female circumcision is a very sensitive issue in Ethiopia and females usually feel ashamed to talk about it. In this study, to reduce the drawbacks of a self-administered questionnaire, detailed information was given to the study participants on the aim of the study, each section of the questionnaire, and their confidentiality. In addition, the authors reduced the questionnaire to a three-point Likert scale to ensure its clarity. Indeed, the data collectors were with the study participants to address any ambiguous questions during the data collection period. The other limitation was the lack of comparability to other studies due to the novelty of the approach.

## Conclusions

In this study, we found that the behavioral intention of primary school girls toward FGC was high. The high intention of school girls towards experiencing FGC implies how much the behavior was deeply-rooted in the community. The study revealed that the intention of respondents toward FGC was a function of perceived barriers and obstacles to overcoming circumcision (direct and indirect perceived behavioral controls), perceived social pressure/subjective norms, mothers’ educational level, and age of the respondents. The findings suggest that FGC is mainly performed by the decision of the parents irrespective of the girls’ preferences. Therefore, designing strategies that empower girls in resisting social pressures and strengthening programs targeted at increasing females’ confidence to confront factors that lead them to circumcision is highly recommended. Interventions on school girls on the other hand help to use them as messengers to educate communities about the negative health implications of female circumcision. Indeed, social and behavioral change communication interventions that target referents have paramount importance. Perhaps, we advise health care providers, women and child affairs, and other concerned bodies to design social and behavioral change communication interventions by focusing on the constructs of the TPB in order to move toward ending FGC. Furthermore, empowering women through education is very important to combat FGC, reduce its negative consequences, and move toward eradication.

## Supporting information

S1 Data (ZIP)

S1 Questionnaire (DOCX)

## Abbreviations

FGC: Female genital cutting
PBC: Perceived behavioral control
SN: Subjective norm
TPB: Theory of planned behavior
