# Supplementary material for: Predictors of uncircumcised primary school girls’ intention to genital cutting in South Ethiopia: Application of theory of planned behavior
Source: PLoS One. 2022 Jun 30;17(6):e0270738. doi: 10.1371/journal.pone.0270738 (PMC9246209; doi:10.1371/journal.pone.0270738)
Supplement: S1 Questionnaire — (DOCX) [file pone.0270738.s002.docx]

**Questionnaire**

**Section I:** **Socio-demographic characteristics of the study participants**

| **S/No** | **Variable** | **Response options** |
| --- | --- | --- |
| 1 | Age in complete years | __________yrs. |
| 2 | Residence | 1. Urban 2. Rural |
| 3 | Students current grade | 1. Grade 5 2. Grade 6 3.Grade 7 4. Grade 8 |
| 4 | Religion | 1. Orthodox 2. Protestant 3.Muslim 4. Others(specify)_________ |
| 5 | Mothers’ educational status | 1. Can’t read and write 2. Can read and write 3. Grade 1-8 4. Grade 9-12 5. Collage and above |
| 6 | Main occupation of the parent | 1. Farmer 2. Employed 3. Merchant 4. Other (specify)____________ |
| 7 | History of sister's circumcision | 1. Yes 2. No |
| 8 | History of circumcision in their neighbors this year | 1. Yes 2. No |

**Section II:** **Behavioural intention toward female genital cutting**

The following items are prepared to measure behavioral intention toward female genital cutting. The response options are: Disagree (D) =1, Neutral (N) =2, and Agree (A) =3

| **S/No** | **Items of behavioral intention** | **Response options** | | |
| --- | --- | --- | --- | --- |
|  |  | **D** | **N** | **A** |
| 1 | I have a willingness to circumcision in the next 1 year |  |  |  |
| 2 | I have a readiness to circumcision in the next 1 year |  |  |  |
| 3 | I have an intention to circumcision in the next 1 year |  |  |  |
| 4 | I will be circumcised in the next 1 year |  |  |  |
| 5 | I want to encourage my friends & sisters to have circumcision |  |  |  |
| 6 | I want to discuss with others on circumcision issue |  |  |  |
| 7 | I have no intention to inform legal bodies if any circumcision attempt is made on me |  |  |  |

**Section III:** **Direct Measures; Constructs of TPB**

The following items are prepared to measure attitude, subjective norm and **perceived behavioral control** toward female genital cutting. The response options are: Disagree (D) =1, Neutral (N) =2, and Agree (A) =3

| **S/No** | **Direct Attitude** | **Response options** | | |
| --- | --- | --- | --- | --- |
|  |  | **D** | **N** | **A** |
| 1 | Female circumcision is good |  |  |  |
| 2 | Female circumcision is important |  |  |  |
| 3 | Female circumcision is pleasant |  |  |  |
|  | **Direct Subjective Norm** |  |  |  |
| 1 | Circumcised girls want me to undergo  circumcision |  |  |  |
| 2 | Most of my referents expect me for having circumcision |  |  |  |
| 3 | Most of my referents want me to experience circumcision |  |  |  |
| 4 | Most people who are important for me inspire for my circumcision |  |  |  |
| 5 | Most people who are important for me like my circumcision |  |  |  |
|  | **Direct Perceived Behavioral Control** |  |  |  |
| 1 | I believe that having circumcision is beyond my control |  |  |  |
| 2 | Resisting circumcision is difficult for  me |  |  |  |
| 3 | I am not fully confident for not having circumcision |  |  |  |

**Section IV:** **Indirect measures; constructs of TPB**

1. **Indirect attitude**

The following items are prepared to measure behavioral beliefs and evaluation of behavior (female genital cutting). The response options are: Disagree (D) =1, Neutral (N) =2, and Agree (A) =3

| **S/No** | **Items of** **behavioral beliefs** | **Response options** | | |
| --- | --- | --- | --- | --- |
|  |  | **D** | **N** | **A** |
| 1 | Female circumcision has no criminal effect |  |  |  |
| 2 | Uncircumcision of female results in feeling of uncleanness |  |  |  |
| 3 | The uncircumcised female has promiscuity behavior |  |  |  |
| 4 | Female circumcision has no health problems |  |  |  |
| 5 | Uncircumcision let female to be insulted by others |  |  |  |
| 6 | Uncircumcision of female is shameful |  |  |  |
| 7 | Uncircumcision is culturally unacceptable |  |  |  |
| 8 | Uncircumcision lead female to be cursed by community |  |  |  |
|  | **Evaluation of behavior** |  |  |  |
| 1 | Criminalization for female circumcision is not good |  |  |  |
| 2 | Circumcision is better than lack of neatness |  |  |  |
| 3 | Circumcision is preferable than having promiscuity |  |  |  |
| 4 | Better to face health problem due to circumcision than staying uncircumcised |  |  |  |
| 5 | Circumcised is preferable to insulted by others |  |  |  |
| 6 | Circumcision is better than feeling shame |  |  |  |
| 7 | Circumcision is good than loose acceptance by the community |  |  |  |
| 8 | Belief in being circumcised than cursed by the community |  |  |  |

1. **Indirect subjective norms**

The following items are prepared to measure normative beliefs and motivations to comply regarding female genital cutting. The response options are: Disagree (D) =1, Neutral (N) =2, and Agree (A) =3

| **S/No** | **Normative beliefs** | **Response options** | | |
| --- | --- | --- | --- | --- |
|  |  | **D** | **N** | **A** |
| 1 | My mother expects me to be circumcised |  |  |  |
| 2 | My father think that I have to be circumcised |  |  |  |
| 3 | My neighbors support my circumcision |  |  |  |
| 4 | My close friends support my circumcision |  |  |  |
| 5 | My sister or brother expect me for having circumcision |  |  |  |
| 6 | My health extension workers expect me for having circumcision |  |  |  |
| 7 | My teachers expect me for having circumcision |  |  |  |
| 8 | Government officials expect me for having circumcision |  |  |  |
| 9 | Uncircumcised girls expect me for circumcision |  |  |  |
| 10 | Circumcised girls expect me for having circumcision |  |  |  |
|  | **Motivations to comply** |  |  |  |
| 1 | My mother approves my circumcision |  |  |  |
| 2 | My father approves my circumcision |  |  |  |
| 3 | My neighbors approve my circumcision |  |  |  |
| 4 | I would like to do what my close friends expect me to do |  |  |  |
| 5 | I would like to do what my sister or brother expects me to do |  |  |  |
| 6 | My health extension workers approve my circumcision |  |  |  |
| 7 | My teachers approve my uncircumcision |  |  |  |
| 8 | I would like to do what government officials expect me to do |  |  |  |
| 9 | Uncircumcised girls approve my circumcision |  |  |  |
| 10 | Circumcised girls approve my circumcision |  |  |  |

1. **Indirect perceived behavioral control**

The following items are prepared to measure Control beliefs and Power of controls regarding female genital cutting. The response options are: Disagree (D) =1, Neutral (N) =2, and Agree (A) =3

| **S/No** | **Control beliefs** | **Response options** | | | |
| --- | --- | --- | --- | --- | --- |
|  |  | **D** | **N** | **A** | |
| 1 | To be uncircumcised, I can’t get the consent of human subjects from family |  |  |  | |
| 2 | Even though I do not want, my family can enforce me to have circumcision |  |  |  | |
| 3 | My family members pre-arrange and schedule for circumcision |  |  |  | |
| 4 | The high prevalence of circumcised girls in my neighborhood has a great influence on me to have circumcision |  |  |  | |
| 5 | Even though the law discourages circumcision, it can’t save me from circumcision |  |  |  | |
| 6 | Communities lack of information about the negative consequences of circumcision putting pressure on me to be circumcised |  |  |  | |
| 7 | High prevalence of circumcised in school girls make my decision hard |  |  |  | |
| 8 | The information I have about the disadvantages of uncircumcision is less likely to save me from circumcision |  |  |  | |
| 9 | My being under family make my decision on circumcision hard |  |  |  | |
|  | **Power of control** |  |  |  |  |
| 1 | My family do not allow me for uncircumcision |  |  |  |  |
| 2 | I fail to resist having circumcision if my family enforced me |  |  |  |  |
| 3 | Pre-arrangement and scheduling of circumcision by my family members makes my decision difficult |  |  |  | |
| 4 | Absence of uncircumcised girls in my neighbor make my decision hard |  |  |  |  |
| 5 | Loose implementation or follow-up of circumcision laws make my decision difficult |  |  |  |  |
| 6 | Communities’ lack of awareness about the consequences of circumcision makes my decision difficult |  |  |  |  |
| 7 | It is difficult to have the power of resisting circumcision due to the high prevalence of circumcised school girls |  |  |  | |
| 8 | The Information I have about circumcision may not realize my decision |  |  |  |  |
| 9 | Being under my family will let me to experience circumcision |  |  |  | |
